# Artemisinin and Its Derivatives from Molecular Mechanisms to Clinical Applications: New Horizons Beyond Antimalarials

**DOI:** 10.3390/ijms26178409

**Published:** 2025-08-29

**Authors:** Yi Xia, Chuanjing Shi, Jingze Lu, Zeyu Zhu, Mohan Li, Yinan Pan, Xinyan Huang, Lei Zhang, Aifen Liu

**Affiliations:** 1 Institute of Interdisciplinary Integrative Medicine Research, School of Medicine, Nantong University, Nantong 226001, China; xiayi@stmail.ntu.edu.cn (Y.X.); shichua0925@stmail.ntu.edu.cn (C.S.); lujingze@stmail.ntu.edu.cn (J.L.); zhuzeyu@stmail.ntu.edu.cn (Z.Z.); limohan@stmail.ntu.edu.cn (M.L.); panyinan@stmail.ntu.edu.cn (Y.P.); huangxinyan@stmail.ntu.edu.cn (X.H.); 2 Department of Pharmaceutical Botany, School of Pharmacy, Naval Medical University, Shanghai 200433, China

**Keywords:** artemisinin, derivatives, antitumor, immunoregulation, metabolic regulation

## Abstract

Artemisinin and its derivatives are widely recognized for their exceptional antimalarial efficacy. Recently, accumulating evidence indicates therapeutic potential beyond malaria. Despite these advances, detailed mechanisms and pharmacological limitations remain incompletely defined. This review summarizes their pharmacological activities and molecular mechanisms associated with oncology, immunoregulation, and metabolic disorders. Mechanistically, these compounds exert potent antitumor effects by inducing oxidative stress, arresting the cell cycle, triggering apoptosis, and inhibiting angiogenesis. They likewise modulate immune responses, re-establishing immune homeostasis and enhancing the effectiveness of immunotherapeutic strategies. Preliminary evidence also suggests involvement in metabolic regulation, pointing to promising avenues for treating metabolic disorders. Given alternative mechanisms of artemisinin and its derivatives, we also discuss the trinity modulation network among antitumor activity, immunoregulation, and metabolic homeostasis. We anticipate that future research will address these knowledge gaps, thereby enhancing the clinical utility of artemisinin and its derivatives and improving patient outcomes across diverse pathologies.

## 1. Introduction

Artemisinin (C_15_H_22_O_5_), a sesquiterpene lactone characterized by a distinctive 1,2,4-trioxane ring, was first isolated through bioassay-guided fractionation in 1972 by Tu Youyou from *Artemisia annua* L. (Asteraceae) [1]. *Atemisia annua* L., a traditional Chinese medicinal herb commonly known as sweet wormwood, stores artemisinin in the glandular secretory trichomes of its leaves. With its bitter and pungent properties, this herb has long been used to relieve summer heat syndrome and treat malaria. In 1981, the World Health Organization and United Nations formally recognized artemisinin’s antimalarial efficacy at the Beijing International Symposium on Antimalarial Drug Development, marking a major milestone that led to the global adoption of artemisinin-based combination therapy (ACTs) as the first-line treatment for *Plasmodium falciparum* malaria [2].

**Figure 1:**
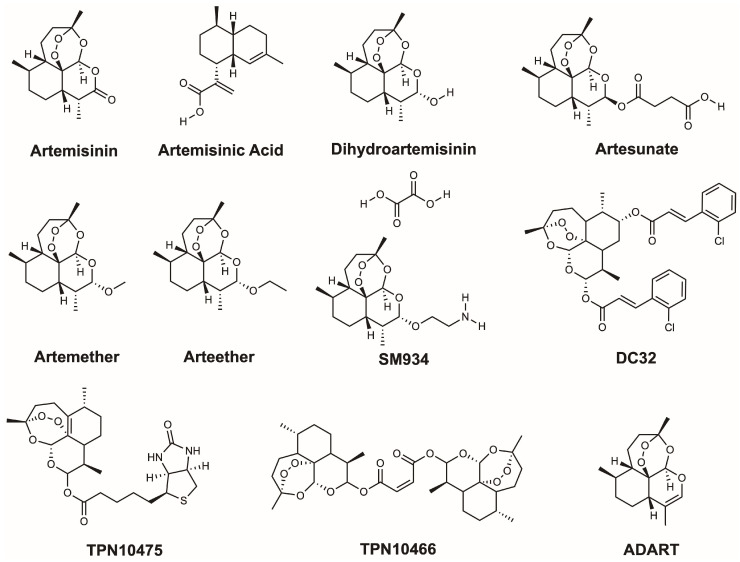
Artemisinin and its derivatives. Artemisinin, biosynthetic precursor (artemisinic acid), biosynthetic intermediate (dihydroartemisinin), core derivatives (artesunate, artemether, arteether), and experimental derivatives (SM934, DC32, TPN10475, TPN10466, ADART). These compounds are drawn in ChemDraw 20.0.

Although artemisinin is the foundational antimalarial compound, it faces significant limitations, including thermal instability, poor solubility, and high production costs. To improve their clinical utility, semi-synthetic derivatives have been developed using artemisinic acid as a precursor to overcome these issues. Among these, dihydroartemisinin (C_15_H_24_O_5_) acts as both a key intermediate and a potent antimalarial agent itself. Furthermore, artesunate (C_19_H_28_O_8_) was designed specifically to provide enhanced water solubility, making it critical for treating severe malaria, while artemether (C_16_H_26_O_5_) and arteether (C_17_H_28_O_5_) utilize their lipophilicity for rapid absorption and efficacy against drug-resistant strains [3,4,5]. To combat emerging resistance, ACTs that rely on these derivatives remain the frontline strategy. Additionally, newer derivatives like SM934 and hybrid molecules not only further optimize pharmacokinetic properties but also expand potential therapeutic applications beyond malaria (Figure 1).

Beyond malaria, growing evidence suggests that artemisinin compounds have therapeutic potential in cancer treatment, immune system regulation, metabolic disorders, and kidney diseases [6,7]. Artemisinin and its derivatives have demonstrated significant antitumor activity against various tumor types (such as lung, breast, colorectal, and liver cancers), and their potential mechanisms (such as inducing ferroptosis, promoting reactive oxygen species production, cell cycle arrest, apoptosis induction, autophagy regulation, and anti-angiogenesis) have been elaborated in several reviews in recent years. However, the current literature has not yet thoroughly elucidated the molecular mechanisms by which ARTs combat tumors, particularly the regulatory network between them and immune regulation, as well as metabolic homeostasis [8,9]. Immunomodulatory effects of dihydroartemisinin have been identified, including its ability to reduce lupus-associated nephritis by inhibiting the production of anti-dsDNA antibodies, reducing TNF-α secretion, and blocking NF-κB signaling [10]. More recently, research has focused on how artemisinin regulates glucose and lipid metabolism via related enzymes and signaling pathways. In addition, clinical trials have been conducted for some diseases, and preliminary results have confirmed their clinical safety and efficacy. However, the exact mechanisms and clinical potential of artemisinin and its derivatives in oncology, immunology, and metabolism are not fully understood. Furthermore, the regulatory network among anti-tumor activity, immune regulation, and metabolic homeostasis requires more in-depth research and discussion.

In this review, we synthesize international research on the antitumor, immunoregulatory, and metabolic effects of probiotics, along with their current clinical applications. Additionally, we explored the interconnected modulation network encompassing antitumor activity, immunoregulation, and metabolic homeostasis, aiming to provide a comprehensive framework for future research and inspire novel therapeutic strategies.

## 2. Antitumor Activity of Artemisinin and Its Derivatives

**Figure 2:**
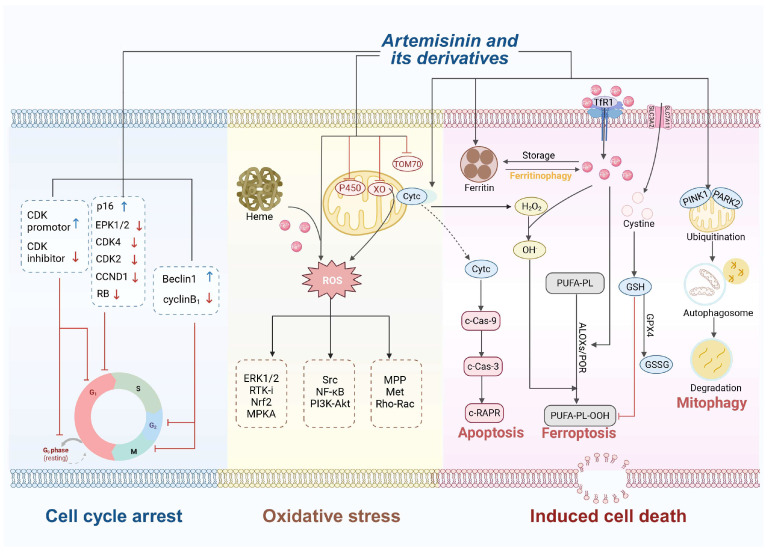
The multifaceted antitumor mechanisms of artemisinin and its derivatives. The mechanisms primarily involve three interconnected biological processes: cell cycle arrest, oxidative stress induction, and the promotion of various forms of cell death. “↓” in black and “↑” in blue denote “enhancing or increasing” while “⊥” and “↓” in red indicate “suppressing or reducing”. This figure is created in BioRender. https://BioRender.com/tea068o (accessed on 21 July 2025).

First reported in 1995, Singh and Lai reported that dihydroartemisinin, in combination with holotransferrin, selectively killed human breast cancer cells in vitro while showing low toxicity to normal cells [11], paving the way for its development as an antitumor agent [12,13]. Subsequent studies have further confirmed its potent anticancer effects, which are mediated through mechanisms such as oxidative stress induction, cell cycle arrest, programmed cell death, and inhibition of angiogenesis (Figure 2).

### 2.1. Mechanisms of Antitumor Activity

#### 2.1.1. Oxidative Stress

Oxidative stress occurs when the production of highly reactive oxygen and nitrogen species during cellular aging or in response to deleterious stimuli exceeds the body’s antioxidant defenses, resulting in tissue damage. Recently, the induction of tumor cell death through oxidative stress has garnered significant attention in cancer therapy [14].

Tumor cells exhibit a dependency on both heme and ferrous iron. Consequently, artemisinin and its derivatives interact with heme-bound Fe^2+^ to produce reactive oxygen species (ROS), which in turn inflict damage to tumor cell membranes, proteins, and DNA [15]. For instance, dihydroartemisinin complexes with transferrin effectively inhibit cancer cell proliferation while sparing normal cells [11,16,17]. These compounds also disrupt mitochondrial function and increase oxidative stress, primarily through peroxisomes and mitochondrial enzymes [18,19]. Specifically, oxidative stress interferes with the electron transport chain in tumor cells, causing electron leakage, reducing membrane potential and ROS production, which triggers mitochondrial dysfunction and apoptosis [20]. For instance, dihydroartemisinin damages mitochondrial DNA and promotes cancer cell death by inhibiting TOM70 [21]. Subsequently, ROS suppresses cancer cell growth, promotes apoptosis, blocks invasion and metastasis, and inhibits angiogenesis by regulating signaling pathways, including ERK1/2, RTK-independent activation, EGFR, and mTOR. This involves key proteins, such as Nrf2 and MAPK, in preventing cancer cell proliferation [22,23]; Src, NF-κB, and PI3K/Akt in promoting cancer cell apoptosis; and matrix metalloproteinases (MMP) secretion, Met overexpression, and Rho-Rac interaction in blocking cancer cell invasion and metastasis. Moreover, ROS regulates VEGF and angiopoietin release during angiogenesis inhibition [24].

#### 2.1.2. Cell Cycle Arrest

The eukaryotic cell cycle, which spans from the end of one cell division to the completion of the next, consists of interphase (G1, S, and G2 phases) and the M phase. This cycle is regulated by genes that encode key proteins, such as cyclins and cyclin-dependent kinases (CDKs) [25].

Artemisinin upregulates p16 and downregulates ERK1/2 and cyclin D1 in gallbladder cancer cells [26]. Halofuginone and artemisinin synergistically arrest cells at G0/G1 by increasing p21 and p27 levels [27]. In hepatoma models, artesunate reduces cyclin D1 and E2F1 while increasing p21 [28]. Some artemisinin compounds induce G2/M arrest. Artesunate triggers cell cycle arrest by inducing cytokinesis defects, multinucleation, centrosome amplification, and multipolar spindle formation [29]. Additionally, it downregulates cyclin B1, enhancing cancer cell radiosensitivity [30], and induces ROS-dependent DNA damage, further blocking cell cycle progression [31].

#### 2.1.3. Induction of Cell Death

##### Apoptosis

Apoptosis is an orderly and autonomous process of genetically controlled cell death that maintains internal environmental stability. Fas activation promotes the mitochondrial release of substances such as cytochrome C, which facilitates the activation of caspases, a key step in apoptosis [32].

Studies on different cell models have shown that artesunate alternatively upregulates Fas and CD95 expression, disrupts mitochondrial membrane potential, releases cytochrome C, and activates caspase-3 and caspase-9, all of which induce apoptosis [33,34]. For instance, artesunate and sorafenib (SOR) synergistically induce apoptosis by significantly upregulating the expression of cleaved caspase-3 and poly (ADP-ribose) polymerase via the STAT3 pathway [35]. Dihydroartemisinin induces the release of cytochrome C and apoptosis-inducing factor from the mitochondria, promotes Bax cleavage, increases caspase-3 levels, and impairs Bcl2 protein expression [36,37]. In addition, artesunate activates p38/MAPK and upregulates the levels of ROS, thereby inducing apoptosis [38,39].

##### Ferroptosis

Ferroptosis, distinct from apoptosis and autophagy, is an iron-dependent programmed cell death. Its mechanisms involve iron accumulation, which catalyzes the lipid peroxidation of polyunsaturated fatty acids (PUFAs) in cell membranes, generating ROS and triggering cell death. Additionally, ferroptosis is driven by the inactivation of GPX4, the central enzyme in the glutathione (GSH)-dependent antioxidant system [40,41].

Artemisinin and its derivatives induce ferroptosis in cancer cells through coordinated modulation of iron metabolism, redox homeostasis, and lipid peroxidation pathways. These compounds trigger ferritinophagy-mediated iron release [42,43] and differentially regulate cellular iron levels. Dihydroartemisinin increases iron accumulation and IRP-IRE binding [42], whereas artesunate decreases iron levels in KTCTL-26 cells [44]. They disrupt the antioxidant defense system by downregulating SLC7A11, GSH, and GPX4 [45,46,47], potentially via p53-dependent mechanisms [48,49]. Concurrently, artemisinin compounds elevate ROS levels via mitochondrial generation [50] and iron-catalyzed reactions [51], while promoting lipid peroxidation through ALOX5 activation [52,53] and the PEBP1/ALOX15 pathway (particularly for dihydroartemisinin) [54]. This multifaceted action leads to irreversible oxidative damage and iron-dependent cell death.

##### Autophagy

Autophagy is a conserved cellular process in eukaryotic organisms, in which damaged proteins and organelles are sequestered by double-membrane vesicles known as autophagosomes. These vesicles subsequently fuse with lysosomes (in animals) or vacuoles (in yeast and plants) to degrade and recycle their contents [55].

Artemisinin and its derivatives exert antitumor effects through multiple forms of autophagy, including mitophagy and ferritinophagy. For example, dihydroartemisinin impairs mitochondrial function, increases ROS production, and activates mitophagy [37,56]. It also facilitates the degradation of ferritin, elevates intracellular iron levels, and enhances ROS accumulation, ultimately driving ferritinophagy [57].

##### Anti-Angiogenesis

Angiogenesis, regulated by diverse factors such as HIF-1α, VEGF, and PDGF, enables endothelial cells to degrade the extracellular matrix and form new blood vessels. This process supplies tumors with oxygen and nutrients and is essential for their progression. Consequently, anti-angiogenic therapies aim to block this process, depriving tumors of critical support and representing a key strategy in cancer treatment [58].

Artemisinin and its derivatives demonstrate potent anti-angiogenic activity by modulating key angiogenic factors across various cancer cell lines [59]. For example, artesunate inhibits the expression of VEGF and PDGF, downregulates HIF-1α, and suppresses vasculogenic mimicry formation, collectively contributing to its antitumor efficacy [60]. Similarly, dihydroartemisinin markedly reduces cancer cell-induced angiogenesis by downregulating the expression of VEGF, MMP-2, and MMP-9 proteins [61].

### 2.2. Applications in Distinct Malignancies

#### 2.2.1. Lung Cancer

Lung cancer remains the leading cause of cancer-related mortality worldwide, with incidence patterns varying by geography and time due to differences in tobacco exposure [62,63,64]. Conventional treatments, including surgery, radiotherapy, and chemotherapy, offer limited efficacy in advanced-stage diseases, underscoring the need for novel therapeutic strategies [65].

Artemisinin and its derivatives have demonstrated antitumor activity in lung cancer [66]. For example, artemisinin suppresses cancer cell proliferation, invasion, differentiation, and angiogenesis by inhibiting sphingosine kinase 1 [67]. Artemisinin treatment reduces CDK4 and COX-2 levels while increasing caspase-3 and IL-6 expression, resulting in apoptosis, cell cycle arrest, and inhibition of microsphere formation in lung cancer cells [68]. Dihydroartemisinin enhances cisplatin sensitivity by inhibiting PTGS1 and activating the ROS–ERK/p38/JNK signaling cascade [69]. It further disrupts tumor progression by blocking ROR1-mediated STAT3 phosphorylation, suppressing HK2/L [70], and TOM70 while activating gasdermin E-mediated pyroptosis [21]. Artesunate binds to FABP5 and inhibits the PPARγ pathway, thereby promoting apoptosis and reducing lung cancer cell proliferation and migration [71]. In non-small cell lung cancer, artesunate also downregulates BTBD7 mRNA expression in a dose-dependent manner, thereby increasing apoptosis and impairing cell migration [72].

#### 2.2.2. Hepatocellular Carcinoma

Hepatocellular carcinoma is the sixth most common cancer worldwide, with approximately 910,000 new cases and 830,000 deaths annually [64,73]. Only about 30% of patients with early-stage disease are eligible for curative surgical resection [74].

Artemisinin and its derivatives exhibit potent antitumor effects in hepatocellular carcinoma (HCC) through diverse molecular mechanisms. Metabolic modulation is a key therapeutic strategy. For instance, these compounds disrupt the YAP1–GLUT1 feedback loop, suppressing glycolysis in tumor cells while enhancing glucose uptake in CD8^+^ T cells to bolster antitumor immunity [75]. Oxidative stress induction further contributes to their efficacy, as dihydroartemisinin triggers ferroptosis by accumulating lipid peroxides and inhibiting the ATF4/xCT antioxidant pathway [76]. Additionally, artemisinin targets transcriptional regulation by downregulating the oncogenic factor FoxM1 to reverse drug resistance in liver cancer cells [77]. Beyond its direct tumoricidal effects, artesunate synergizes with sorafenib (SOR) by activating the AFAP1L2–SRC–FUNDC1 axis, thereby promoting mitochondrial autophagy and amplifying therapeutic outcomes [78].

#### 2.2.3. Breast Cancer

Breast cancer is the most commonly diagnosed malignancy among women, accounting for approximately 2.3 million new cases annually [64]. While early-stage patients benefit from over 90% five-year survival rates with surgery and radiotherapy [79], late-stage breast cancer remains challenging, with observed chemotherapy resistance [80].

Artemisinin and its derivatives have overcome breast cancer drug resistance by inducing ferroptosis and regulating autophagy–apoptosis pathways [81]. For example, artemisinin suppresses migration and chemoresistance in LCC9 cells by modulating the expression of resistance-related genes [58]. Dihydroartemisinin also promotes ferroptosis in breast cancer by downregulating hsa_circ_0001610 and enhancing radiosensitivity [82]. Furthermore, artesunate induces apoptosis by modulating Bax/Bcl-2 and G0/G1 cell cycle arrest by regulating IGF-IR levels in MCF-7 cells [83]. However, the clinical efficacy of artemisinin-based therapies is limited by their poor tumor-targeting capabilities. To address this issue, a glutathione-sensitive artemisinin ester conjugate in liposomes (TPP-SS-ATS-LS) was encapsulated to target both tumor cells and mitochondria. This delivery system significantly enhances antitumor activity in breast cancer mouse models, increasing tumor growth inhibition from 37.7% to 56.4% [84].

#### 2.2.4. Colorectal Cancer

Colorectal cancer accounts for approximately 1.93 million new cases annually worldwide, with its incidence rising notably in developing countries [64,85]. Early-stage patients benefit from a 70–80% five-year survival rate with surgical resection, while outcomes for advanced-stage patients remain poor [86].

Emerging evidence suggests that artemisinin and its derivatives have therapeutic potential in colorectal cancer through multiple mechanisms, including the induction of apoptosis, inhibition of angiogenesis, and modulation of the tumor immune microenvironment [87]. For example, artemisinin has been shown to bind to microsomal prostaglandin E synthase-2 (mPGES-2), thereby reducing PGE2 production and suppressing colorectal cancer cell proliferation [88]. Dihydroartemisinin, a key derivative, inhibits glycogen synthase kinase 3β (GSK-3β), disrupts the TCF7/MMP9 signaling pathway, and promotes β-catenin degradation, consequently impairing tumor growth and metastasis. Moreover, it displays synergistic effects when combined with capecitabine, enhancing antitumor immunity and improving efficacy while alleviating chemotherapy-associated side effects [89].

#### 2.2.5. Other Tumors

Artemisinin and its derivatives have also demonstrated antitumor activity against a variety of malignancies, including leukemia, ovarian cancer, glioblastoma, prostate cancer, and melanoma, as summarized in Table 1. These mechanisms include the induction of oxidative stress, cell cycle arrest, apoptosis, autophagy, and inhibition of angiogenesis.

## 3. Immunomodulatory Activity of Artemisinin and Its Derivatives

**Figure 3:**
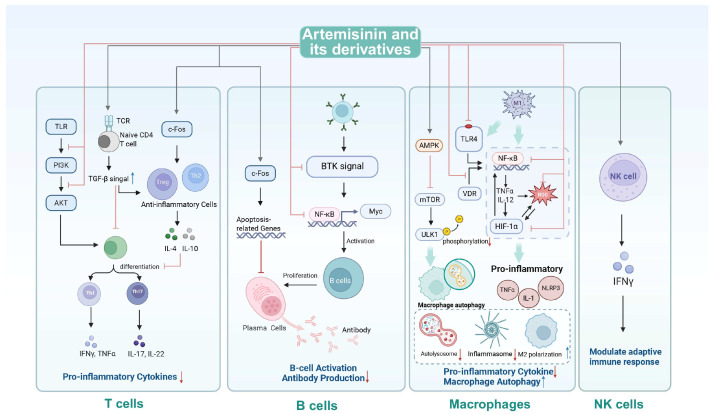
The diverse immune regulation of artemisinin and its derivatives. The immunoregulatory actions of artemisinin and its derivatives are explored through the lens of distinct immune cell types, specifically T cells, B cells, macrophages, and natural killer (NK) cells. “↓” in black and “↑” in blue denote “enhancing or increasing” while “⊥” and “↓” in red indicate “suppressing or reducing”. This figure is created in BioRender. https://BioRender.com/9j6k8fd (accessed on 21 July 2025).

Artemisinin and its derivatives, initially studied for immunomodulatory effects in the early 2000s, showed promise in treating autoimmune and inflammatory diseases while ensuring safety. Recent findings have revealed their ability to modulate vital immune cells, including T cells, B cells, macrophages, and NK cells (Figure 3).

### 3.1. Mechanisms of Immunomodulatory Activity

#### 3.1.1. T-Cell Regulation

T lymphocytes play a central role in maintaining immune homeostasis by coordinating cellular and humoral immune responses. They help regulate B cell–mediated antibody production through the secretion of various cytokines [121].

Artemisinin and its derivatives exhibit broad immunomodulatory effects by targeting multiple aspects of T and B cell function. Specifically, artemisinin suppresses pro-inflammatory T cell activity by inhibiting the proliferation of inflammatory subsets (e.g., Th1 and Th17) and downregulating key cytokines, such as IL-2 and IFN-γ [122,123]. These effects are mediated by the inhibition of critical signaling pathways, including Ras-Raf1-ERK1/2 [124], PI3K-AKT [125,126], and TLR [127] in T cells, which collectively impair T cell activation and differentiation. For instance, TPN10475 counteracts TCR-induced suppression of TGF-β signaling, further inhibiting effector CD4^+^ T cell function in vitro [128]. Additionally, artemisinin modulates regulatory T cells (Tregs) by upregulating their populations [122,123], a process driven by dihydroartemisinin’s activation of the c-Fos transcription factor, which enhances expression of proliferation-related genes (e.g., VEGF, T cell receptor, IL-2) [129]. By simultaneously suppressing inflammatory T cell responses and promoting Treg-mediated tolerance, artemisinin compounds effectively restore immune homeostasis and indirectly influence B cell-driven humoral immunity through T cell cytokine regulation [121].

#### 3.1.2. B-Cell Regulation

B lymphocytes play a crucial role in adaptive immunity by mediating antigen-specific and humoral responses. Through the production of antibodies, they orchestrate pathogen neutralization, opsonization, and complement activation while simultaneously presenting antigens to T cells to bridge innate and adaptive immunity.

Artemisinin and its derivatives significantly modulate humoral immunity by targeting B lymphocytes’ function through multiple mechanisms. First, these compounds regulate B cell activation and plasma cell (PC) differentiation, ultimately leading to the suppression of B cell-dependent IgM antibody production [130]. Dihydroartemisinin blocks Bruton’s tyrosine kinase signaling and its downstream NF-κB and Myc pathways, directly inhibiting B cell activation and differentiation, and decreasing the number of splenic and circulatory B cells, thereby suppressing B cell response and antibody production in systemic lupus erythematosus [131,132]. Artesunate inhibits the BAFF-induced NF-κB signaling pathway by regulating the ubiquitination and degradation of TRAF6. This restricts the overactivation, proliferation, and survival of B cells and decreases the infiltration of lymphocytes and secretion of autoantibodies [133]. TRAF6 also serves as a critical adaptor in BAFF-mediated B cell signaling. Consequently, artesunate is expected to restrict excessive B-cell activation, proliferation, and survival. These compounds inhibit the proliferation of splenic marginal zone B cells while increasing the relative proportion of CD21 ^(low)^ CD23^(+)^ follicular and B10 B cells. This shift negatively regulates IL-10 secretion, thereby weakening B cell-mediated immune responses [134]. Collectively, these results indicate potential clinical applications for attenuating antibody-mediated rejection (ABMR).

#### 3.1.3. Macrophage Modulation

Macrophages play essential roles in both cellular and humoral immunity, acting as antigen-presenting and effector cells, and engaging in the phagocytosis of pathogens. During both acute and chronic inflammatory responses, macrophages secrete proinflammatory cytokines through pathways such as NF-κB activation [135].

Artemisinin and its derivatives modulate macrophage activity by suppressing pro-inflammatory cytokine production and enhancing macrophage autophagy. For instance, chitosan-coated artesunate inhibits TLR4/NF-κB signaling and promotes STAT6-driven M2 macrophage reprogramming in ulcerative colitis [136]. Artesunate blocks TLR4-dependent autophagy in acute pancreatitis while activating the HIF-1α/NF-κB signaling cascade to drive macrophage repolarization in rheumatoid arthritis [137,138]. Dihydroartemisinin blocks TLR4-mediated activation of NF-κB-STAT3/MAPK cascades, ameliorating the innate inflammatory response induced by muramidase-released protein [139]. SM934 inhibits the TLR4/NF-κB/NLRP3 axis in dry eye disease [140]. Artemisinin also enhances M2 polarization by downregulating the MYD88/ERK signaling [141]. Concurrently, it activates the JNK/MAPK-caspase-9 apoptotic pathway, further suppressing pro-inflammatory cytokines (IL-12, TNF-α), and demonstrates therapeutic efficacy in colitis [142]. Collectively, these mechanisms reduce pro-inflammatory cytokine expression, inhibit M1-type polarization, and promote M2-type macrophage reprogramming.

#### 3.1.4. Natural Killer Cell Potentiation

Natural killer (NK) cells are pivotal innate immune effectors that directly eliminate virus-infected and tumor cells. They concurrently secrete cytokines, such as interferon-γ, to enhance immune surveillance and clearance and to modulate the adaptive immune response. Pretreatment with artemisinin and its derivatives sensitizes tumor cells to NK cell-mediated killing in vitro by increasing the binding of NK cells to target cells and upregulating the expression of apoptosis-related genes in tumor cells [143,144]. These mechanisms enhance susceptibility to NK cell cytotoxicity, thereby amplifying the immunological efficacy of NK cells.

### 3.2. Applications in Immune-Mediated Disorders

#### 3.2.1. Systemic Lupus Erythematosus

Systemic lupus erythematosus (SLE), a multi-organ autoimmune disease, exhibits significant geographic variations in its global prevalence [145,146]. Although glucocorticoids and immunosuppressants have been shown to significantly alleviate symptoms, their therapeutic efficacy remains limited, particularly in severe cases refractory to conventional treatments [147,148].

Artemisinin and its derivatives exhibit immunomodulatory effects in SLE by restoring immune cell homeostasis and suppressing inflammatory responses. Dihydroartemisinin has been shown to inhibit T follicular helper (Tfh) and B cells, reduce abnormal antibodies [129], and restore Treg/Th17 balance by upregulating Treg cells and downregulating Th17 cells, thereby mitigating excessive immune responses and autoimmune tissue damage in SLE [149]. Artesunate reduces serum ANA and anti-dsDNA titers, urinary protein, and creatinine levels in SLE-model mice. It lowers MCP-1 levels in serum, urine, and kidneys and decreases BAFF expression in the spleen, thereby modulating the immune system and improving SLE [150]. Furthermore, SM934 demonstrates favorable efficacy in the lupus-prone female MRL/lpr mouse model, and its mechanism of action is mainly reflected in the regulation of T cell subsets. In vitro, SM934 inhibits IFN-γ and IL-17 production by activating CD4^+^ T cells and suppresses their differentiation into Th1 and Th17 cells. In vivo, it increases Treg levels, inhibits Th1 and Th17 development, and blocks STAT1, STAT3, and STAT5 activation in splenocytes [151].

#### 3.2.2. Rheumatoid Arthritis

Rheumatoid arthritis (RA) is characterized by synovial inflammation and progressive bone erosion [152]. Despite progress in RA treatment, some refractory cases, particularly seronegative conditions, continue to pose a risk of irreversible joint damage, underscoring the need for optimized drug efficacy and safety [153].

Emerging evidence has demonstrated the potential of artemisinin derivatives for RA treatment. For instance, DC32, a dihydroartemisinin derivative, ameliorates footpad swelling, cartilage degradation, and inflammatory cell infiltration in a collagen-induced arthritis (CIA) model via the Nrf2-p62-Keap1 feedback loop [154]. Artemisinin shows positive effects in a CIA model in *DBA*/1J mice by inhibiting RA-FLS proliferation, inducing apoptosis, and reducing migration and invasion [155]. SM934 inhibits Tfh and Th17 cell development and suppresses CII-reactive T cell proliferation and inflammatory cytokine secretion in *DBA*/1J mice, thereby limiting pathogenic antibody production. In vitro, it also inhibits naïve CD4^+^ T cell polarization to Tfh, Bcl-6 expression, IL-21-producing CD4^+^ T cells, and STAT3 signaling, thereby regulating immune responses [156]. In addition, artesunate affects the Th17/Treg lymphocyte balance by regulating Treg cell apoptosis and Th17 cell proliferation [157].

#### 3.2.3. Psoriasis

Psoriasis is an immune-mediated disease that affects the skin and joints. Although a multilevel therapeutic regimen has been developed (consisting of topical retinoids, photochemotherapy, bio-targeted agents, and small-molecule inhibitors), treatment resistance persists in specific subtypes, such as recalcitrant plaque lesions and generalized pustular psoriasis [158].

Artemisinin and its derivatives have demonstrated significant therapeutic potential in both animal models and preclinical studies. For example, dihydroartemisinin alleviates skin inflammation by modulating T cell subsets, suppressing cytokine levels, and inhibiting NF-κB phosphorylation and key transcription factors in psoriasis [159]. Artemisinin also modulates immune responses by reducing γδ T cell populations in lymph nodes without affecting Th17 cells, thereby lowering serum IFN-γ and IL-17 levels and suppressing keratinocyte proliferation and epidermal thickening [160]. Additionally, artesunate inhibits TNFα–induced IL-23 expression in HaCaT keratinocyte cells, a key driver of inflammatory cell recruitment and activation in psoriatic lesions [161].

#### 3.2.4. Multiple Sclerosis

Multiple sclerosis (MS) progressively destroys myelin sheaths in the central nervous system and shows sex and latitudinal incidence variations. Despite modern treatments like β-interferon and B-cell depletion, effective strategies to prevent neurodegeneration in primary progressive MS are lacking, making neural axon repair and nerve function restoration challenging, particularly in cases of severe fatigue or cognitive dysfunction [162].

Artemisinin and its derivatives primarily treat MS by regulating immune cell activity and preserving myelin integrity. In experimental autoimmune EAE mouse models, they suppress IFN-γ production while promoting IL-4 expression, effectively shifting the immune response from Th1 to Th2 responses. This immune polarization reduces Th1-mediated inflammation and alleviates disease severity, although the precise mechanisms remain unclear [163,164]. Additionally, the artemisinin derivative ADART exhibits potent efficacy in MS models, inhibiting IFN-γ and IL-17 production in vitro, reducing the ratios of Th1 and Th17 cells, and lowering the levels of IFN-γ and IL-17A in vivo, further contributing to disease attenuation [165].

#### 3.2.5. Other Immune Disorders

Artemisinin and its derivatives have also demonstrated efficacy on other immune diseases, including transplant rejection, type 1 diabetes, autoimmune hepatitis, experimental autoimmune encephalomyelitis, and melanoma (Table 2). These effects include promoting T and B cell proliferation, regulating the release of cytokines, and modulating signaling pathways.

## 4. Metabolic Regulation of Artemisinin and Its Derivatives

**Figure 4:**
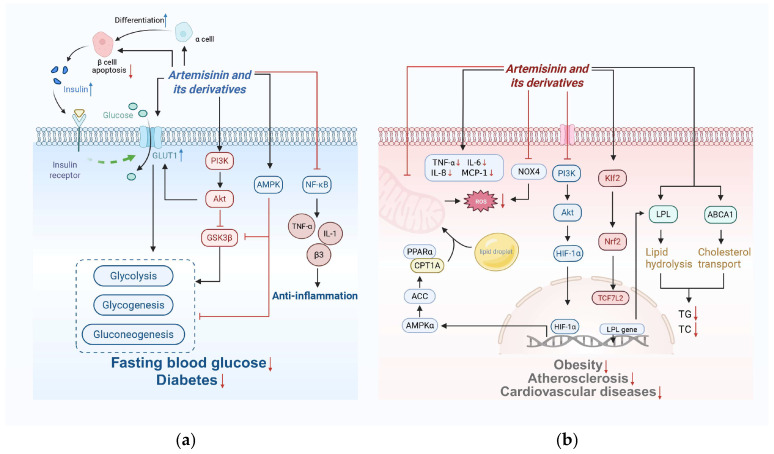
The diverse immune regulation of artemisinin and its derivatives. (**a**) The mechanisms of artemisinin and its derivatives on glucose metabolism; (**b**) The mechanisms of artemisinin and its derivatives on lipid metabolism. “↓” in black and “↑” in blue denote “enhancing or increasing” while “⊥” and “↓” in red indicate “suppressing or reducing”. This figure is created in BioRender. https://BioRender.com/m0zxmmb (accessed on 21 July 2025).

Artemisinin and its derivatives regulate glucose and lipid metabolism through key enzymes, altering intracellular signaling pathways, and counteracting oxidative stress (Figure 4). Recent work has extended these findings to metabolic diseases, such as diabetes, obesity, and atherosclerosis.

### 4.1. Glucose Metabolism

Glucose metabolism is intricately regulated by a multitude of factors, such as hormonal regulation, rate-limiting enzymes within glycolysis, allosteric modulation, and gene expression regulation. Scholarly interest in the impact of artemisinin and its derivatives on glucose metabolism has been increasing, especially in the context of diabetes, which represents a significant global health issue [176,177]. Their antidiabetic effects involve activating the insulin signaling pathway [178], enhancing glucose transport [179], and improving both intracellular glucose metabolism and inflammation [119].

Artemisinin and its derivatives activate the insulin signaling pathway, thereby promoting glucose uptake and utilization in cells [178]. They also improve insulin resistance by reducing pancreatic β-cell apoptosis, enhancing β-cell function, and increasing insulin secretion. In db/db mice, artemether significantly improves insulin sensitivity and glucose homeostasis [180]. Additionally, artemisinin promotes the transdifferentiation of pancreatic α-cells into β-cells [181]. This effect may be mediated by GABAA receptor signaling, although this mechanism remains controversial and requires further investigation [182]. Artemisinin also plays a therapeutic role in type 2 diabetes by enhancing GLUT1 expression, which further promotes glucose metabolism [183]. Additionally, novel artemisinin derivatives target GLUT1, affecting tumor cell metabolism [179,184].

Artemisinin and its derivatives inhibit glycogen synthase and improve intracellular glucose metabolism and inflammatory responses through multiple signaling pathways. AMPK, a key metabolism-regulating enzyme, regulates the metabolism of glucose, lipids, and proteins. Artemisinin promotes glucose uptake and utilization in mice with diabetes by activating the AMPK pathway while inhibiting the activity of GSK-3β, thus improving glucose tolerance and insulin sensitivity [89]. Furthermore, it also affects multiple signaling pathways, including TGF-β [185], β3-integrin [133], and RANKL [186], and influences ERK [187,188,189], PI3K, and AKT [183,190], which regulate glucose metabolism and inflammation. Additionally, they inhibit NF-κB activity, leading to the downregulation of inflammatory cytokines and indirectly disrupting glucose metabolism [191].

Regarding clinical application potential, artemisinin and its derivatives have shown promising efficacy in diabetes-related studies. They not only significantly reduce blood glucose levels but also provide protection against diabetes-related complications, such as diabetic cardiomyopathy, diabetic retinopathy, diabetic nephropathy, and diabetes-induced nerve damage [185,192,193].

### 4.2. Lipid Metabolism

Artemisinin and its derivatives modulate lipid metabolism by inhibiting lipogenesis, promoting lipid degradation, and restoring cholesterol metabolism homeostasis. They therefore show therapeutic potential for diseases such as obesity, atherosclerosis, and cardiovascular diseases.

Regarding the suppression of lipogenesis, artemisinin and its derivatives significantly reduced fat accumulation and improved lipid profiles in high-fat diet-induced mouse models by regulating key signaling pathways. Research has demonstrated that the PPAR signaling pathway plays a pivotal role in this process, primarily by reducing the phosphorylation levels of PPAR-γ and C/EBP-α [194,195]. Concurrently, these compounds effectively inhibit adipocyte differentiation and lipid storage by suppressing the Akt/mTOR signaling pathway [196].

Artemether exhibits remarkable efficacy in promoting lipolysis and energy metabolism. It activates the p38 MAPK/ATF2 signaling pathway to induce white adipose tissue browning and enhance brown adipose tissue thermogenesis, thereby increasing energy expenditure [196]. Furthermore, in cholesterol metabolism regulation, artemisinin upregulates Klf2 expression, facilitating the binding of TCF7L2 to the LPL promoter, which subsequently increases LPL expression and promotes lipid hydrolysis, ultimately reducing plasma cholesterol and triglyceride levels [172,182]. Artesunate has demonstrated significant lipid-lowering effects. Preclinical studies have shown that this compound ameliorates aortic root lesions and reduces body weight in obesity models without causing adverse effects, such as nausea [197]. This effect is closely associated with the ability to regulate cholesterol homeostasis.

With respect to anti-inflammatory and antioxidant effects, artemisinin effectively inhibits ROS production by downregulating NOX4 expression in adipocytes [198,199,200], thereby restricting adipocyte differentiation and lipid storage, ultimately reducing fat mass and improving glucose-lipid metabolism in obese mice [200]. Notably, in atherosclerosis models, artemisinin and its derivatives significantly attenuate plaque formation and improve vascular health by preventing lipid accumulation, alleviating oxidative stress, and exerting anti-inflammatory effects [201,202]. Specifically, artesunate reduces the expression levels of pro-inflammatory cytokines (TNF-α, IL-6) and chemokines (IL-8, MCP-1) [194], while simultaneously inhibiting M1 macrophage polarization and lipid deposition in vascular walls [203].

Taken together, these findings highlight the potential of artemisinin and its derivatives as therapeutic agents for lipid metabolism disorders, especially obesity and atherosclerosis (Table 3).

### 4.3. Regulation of Oxidative Stress and Metabolism Interplay

Artemisinin and its derivatives exert protective effects by simultaneously targeting the dysregulated interplay between oxidative stress and metabolic dysfunction, particularly in glucose and lipid metabolism. Unlike their pro-oxidant actions in tumors, these compounds alleviate oxidative stress in metabolic contexts via three interconnected mechanisms.

Artemisinin and its derivatives activate integrated antioxidant-metabolic defenses. They initiate protection via the Nrf2 pathway activation. This upregulates antioxidant enzymes (SOD, GSH), directly reducing oxidative damage in pathologies like diabetic nephropathy [187,211,214,215]. Significantly, this mechanism intersects with metabolic regulation by concurrently supporting lipid homeostasis [194,200,207], demonstrating intrinsic crosstalk between antioxidant and metabolic systems.

Disruption of inflammatory oxidative amplification cycles represents the second axis of intervention. Elevated ROS levels trigger NF-κB-mediated release of cytokines (TNF-α, IL-6), which further stimulates ROS production [216,217]. By suppressing NF-κB activation, artemisinin reduces cytokine levels (TNF-α, IL-6, IL-8, and MCP-1), thereby interrupting this vicious cycle. Crucially, this anti-inflammatory action translates to improved metabolic outcomes, as evidenced by restored glucose homeostasis in diabetes [218], directly linking oxidative stress control to metabolic benefits.

Targeting metabolic origins of oxidative stress completes the regulatory triad. Hyperglycemia induces oxidative stress via the polyol pathway, formation of advanced glycation end products (AGEs), and activation of protein kinase C (PKC), all of which impair insulin sensitivity and β-cell function [219,220]. Artemisinin compounds enhance insulin signaling and glucose uptake, partly by activating the MAPK and PI3K/AKT pathways [221,222]. Parallelly, it ameliorates lipid-driven oxidative stress caused by fatty acid accumulation, mitochondrial ROS, and NOX4 activation [200], through NOX4 downregulation and PPARs/Akt/mTOR modulation [194,200,207]. Moreover, artemisinin activates AMPK signaling, protecting against oxidative damage by agents like amiodarone [209,210].

In summary, artemisinin and its derivatives combat metabolic oxidative stress through multiple coordinated mechanisms, activating antioxidant pathways and improving both glucose and lipid metabolism. These multifaceted effects not only alleviate symptoms but also target upstream drivers of disease, highlighting their promising clinical potential.

## 5. The Trinity Modulation Network Among Antitumor Activity, Immunoregulation and Metabolic Homeostasis

### 5.1. Antitumor-Immunometabolic Synergy

Immunotherapy has emerged as a cornerstone of cancer treatment, mobilizing the host’s adaptive and innate immune systems against tumors [223,224]. The overlapping metabolic reprogramming of cancer and immune cells is now recognized as a pivotal determinant of antitumor immunity [225,226]. Within this framework, artemisinin compounds demonstrate dual targeting of immunometabolic crosstalk. For instance, dihydroartemisinin and artemether disrupt tumor aerobic glycolysis in non-small cell lung cancer by suppressing ERK/c-Myc signaling [227], while concurrently reprogramming immune responses—as evidenced by dihydroartemisinin delaying head and neck squamous cell carcinoma progression through reduced M2-TAM infiltration and angiogenesis inhibition [228]. Critically, this metabolic interference promotes immunostimulatory monocyte-to-dendritic cell differentiation while counteracting immunosuppressive pathways [227], establishing a mechanistic synergy. Thus, the convergence of artemisinin-induced metabolic modulation and immune activation provides a compelling rationale for combining these agents with immunotherapies targeting the immunometabolic network [226], thereby potentiating antitumor efficacy.

### 5.2. Oxidative Stress Regulation in Tumor and Metabolic Diseases

As mentioned in Section 3.1.1 and Section 5.3, artemisinin and its derivatives show promising therapeutic potential for regulating oxidative stress based on the microenvironment. In tumors, they promote ROS generation via the Fenton reaction in an iron-rich environment [22,26], leading to ferroptosis and mitochondrial permeability transition [21]. Although Nrf2 activation aids tumor survival under normal conditions, combining it with pro-oxidative therapies can induce synthetic lethality at elevated ROS levels. Conversely, in metabolic disorders, artemisinin and its derivatives protect against metabolic disorders by reducing NOX4-derived ROS, enhancing insulin/PI3K/AKT signaling, and blocking NF-κB/NLRP3 inflammatory pathways [183,229,230], shifting mitochondria from ROS amplifying in tumors to scavenging in metabolic disorders. Moreover, therapeutic strategies differ. Tumors focus on inhibiting angiogenesis and metastasis, while metabolic reprogramming emphasizes lipid oxidation and detoxification [231,232].

These approaches reflect the distinct microenvironments involved in tumor and metabolic disease development. Tumors rely on iron metabolism for ROS buildup, while metabolic disorders need ROS clearance [233]. Despite these differences, both strategies converge on mitochondrial regulation, underscoring the key role of organelles in redox adaptation. Given Nrf2’s dual role, tissue-targeted delivery systems are essential. Artemisinin and its derivatives offer a versatile platform for combination therapies targeting tumor cytotoxicity and metabolic balance.

### 5.3. Immune-Metabolic Crosstalk

Immune cell function is intrinsically linked to metabolic reprogramming, and redox metabolism plays a pivotal role in maintaining internal homeostasis. Antioxidant systems, such as thioredoxin (TRX) and glutathione (GSH), regulate the proliferation and function of T cells, B cells, and macrophages primarily through molecular hubs like Nrf2 [234,235]. Within this framework, artemisinin and its derivatives exert targeted immunomodulatory effects by reprogramming cellular metabolism. They inhibit pro-inflammatory M1 macrophage polarization while promoting anti-inflammatory M2 phenotypes via suppression of the NF-κB/NLRP3 pathway [230]. Furthermore, artemisinin critically disrupts T-cell differentiation by altering metabolic preferences. Th17 cells (pro-inflammatory) depend on glycolysis and glutamine metabolism, whereas Treg cells (anti-inflammatory) rely on oxidative phosphorylation and amino acid catabolism [236]. By activating AMPK and inhibiting mTORC1 signaling [107], artemisinin compounds shift T cell metabolism from glycolysis to fatty acid oxidation, thereby suppressing Th17 differentiation and promoting Treg expansion [237].

This crosstalk extends to metabolic pathologies in which immune dysfunction exacerbates disease progression. In atherosclerosis, inflammatory cytokines (IL-1β, TNF-α) and M1 macrophages drive plaque inflammation, necrosis, and thrombosis [238]. Similarly, Th17/Treg imbalance perpetuates chronic inflammation in obesity and in type 2 diabetes [239]. Artemisinin counteracts these processes through two mechanisms: first, by suppressing the NF-κB/NLRP3 inflammasome to reduce caspase-1, IL-1β, and TGF-β levels [96,230]; and second, by restoring Th17/Treg equilibrium through metabolic reprogramming [230]. Consequently, the simultaneous targeting of the immune and metabolic axes by artemisinin provides novel therapeutic implications for immunotherapy in metabolic disorders.

## 6. Clinical Safety and Efficacy

ACTs demonstrate subtype-specific clinical efficacy and safety when administered according to the WHO-recommended regimens [240]. For uncomplicated *Plasmodium vivax* or *ovale* malaria, standard ACT regimens, including dihydroartemisinin-piperaquine or artesunate-amodiaquine, achieve clinical efficacy when combined with primaquine at total doses of either 180 mg administered over 8 days or 210 mg over 14 days. These regimens show no reported severe adverse events, even in cases of mixed infections. In *Plasmodium malariae* or *knowlesi* malaria, abbreviated ACT regimens have similar efficacy and safety profiles. Effective options include dihydroartemisinin-piperaquine administered with an initial loading dose, followed by fractional dosing; artesunate-amodiaquine given as two tablets daily for three consecutive days; or a single 8-tablet dose of artemether-naphthoquine. For severe malaria cases, intravenous artesunate remains the first-line therapy due to its superior hemodynamic stability compared to intramuscular artemether. The recommended protocol for adults involves administration of 120 mg doses at 0, 12, and 24 h, followed by a daily maintenance dose. Pediatric patients weighing less than 20 kg should receive 3 mg/kg per dose. Clinicians should exercise particular caution when treating patients presenting with shock. Once patients regain consciousness and demonstrate oral tolerance, a transition to oral ACTs is clinically appropriate.

Artemisinin and its derivatives have shown preliminary anticancer efficacy with favorable safety profiles in early clinical trials across multiple malignancies. In breast cancer, Phase I/II clinical trials, including NCT00764036, have evaluated oral artemether at doses of 100, 150, or 200 mg daily, showing preliminary anticancer efficacy with good safety and tolerability, although no Phase III trials have been initiated [241]. Similarly, in colorectal cancer, a randomized, double-blind trial (*n* = 23) reported that oral artemisinin increased tumor apoptosis (>7%) without significant adverse events, supporting its pro-apoptotic mechanism observed in preclinical studies [242]. For cervical cancer, research has progressed to Phase I trials assessing intravaginal artesunate and oral dihydroartemisinin formulations [243]. In summary, artemisinin and its derivatives offer several potential advantages, including broad-spectrum anticancer activity, minimal adverse reactions in clinical trials, and no cross-resistance with conventional therapies. However, there are still significant limitations, as most evidence comes from small-scale early-phase trials or preclinical studies. Future research should conduct larger, well-designed Phase II/III trials to validate clinical efficacy and establish long-term safety profiles, particularly for the extended treatment durations required in oncology compared with malaria applications [244].

In immune system diseases, artemisinin and its derivatives have demonstrated promising clinical efficacy for SLE treatment. An early randomized controlled trial involving 45 SLE patients showed that artemisinin tablets administered at 50 mg twice daily combined with prednisone at 0.25 mg to 0.8 mg per kilogram per day significantly reduced disease activity and improved symptoms including fever, joint pain, erythema, rash, and hair loss compared to only prednisone at 0.8 mg to 1.25 mg per kilogram per day, with comparable safety profiles [245]. Advancing clinical development further, SM934 is approved as a Class I new drug by the China National Medical Products Administration in 2015 and is currently undergoing Phase II clinical trials. Preclinical studies have shown that it has superior efficacy in alleviating kidney and cutaneous lupus lesions, with lower toxicity than traditional immunosuppressants like cyclosporine A [246]. Although these preliminary findings are promising, larger Phase II and III trials are needed to fully establish the long-term efficacy and safety of these compounds in SLE treatment.

Current preclinical research on artemisinin and its derivatives in metabolic diseases has mainly focused on diabetes and its complications, obesity-related metabolic disorders, hyperuricemic nephropathy, and non-alcoholic fatty liver disease. However, to date, no clinical trials have been conducted. The optimal dosing parameters, administration protocol, and comprehensive safety assessments still need further investigation prior to clinical translation.

In summary, compared to the well-established short-course antimalarial treatments, artemisinin and its derivatives require relatively long treatment cycles and/or high-intensity exposure to achieve efficacy in oncology, immunomodulation, and metabolism [247]. However, this may increase the risk of mechanism-related toxicities. Artemisinin derivatives exhibit significant dose- and time-dependent differences in various therapeutic applications, inevitably triggering the inherent mechanism-based toxicity risks of the drug, including neurotoxicity, cardiotoxicity, embryotoxicity, and hematopoietic suppression, etc. [248]. Based on the above analysis, the dose-time-dependent toxicity risk of artemisinin-based drugs should be prioritized in clinical translation.

## 7. Conclusions and Outlook

Artemisinin and its derivatives are not only highly effective in treating malaria but also show potential in cancer therapy, immune regulation, and metabolic disorders. In cancer, they induce apoptosis, inhibit angiogenesis, and arrest the cell cycle. Importantly, they overcome drug resistance to traditional chemotherapies [244]. In immunoregulation, they enhance immune responses and macrophage activity [249]. Furthermore, in metabolic regulation, they help regulate blood sugar and lipid levels, offering new treatment options for metabolic diseases.

Although artemisinin and its derivatives share fundamental anticancer properties due to the structures of peroxide bridge-mediated free radical generation and ferroptosis induction, there are several important mechanisms in different tumor types. Firstly, the tumor selectivity of artemisinin derivatives depends on their structural modifications [250] and the intracellular levels of labile ferrous iron [42]. Artesunate C-10 succinate enhances aqueous solubility and enables intravenous administration. Its carboxyl group mediates transferrin binding, selectively targeting TfR-high cancer cells in HCC [28]. The lipophilic ether bond of artemether confers selective penetration advantages for treating breast cancer and gliomas by enhancing blood-brain barrier and tumor membrane permeability [251]. Second, the target specificity and mechanistic selectivity of artemisinin derivatives are closely associated with distinct oncogenic driver pathways across different tumor types. In HCC, artemisinin disrupts cellular bioenergetics and inactivates Hippo-YAP signaling [252], while artesunate exacerbates AFAP1L2-SRC-FUNDC1 axis-dependent mitophagy and mitigates sorafenib resistance [78]. For breast cancer, artemisinin derivatives suppress TGF-β signaling and inactivate cancer-associated fibroblasts [253]. Finally, distinct immunomodulatory effects arise from tumor microenvironment (TME) heterogeneity. For example, hepatocellular carcinoma primarily relies on innate immune regulation (macrophages) [254], while breast and lung cancers emphasize adaptive immunity (T cells/NK cells) [255,256]. Overall, artemisinin derivatives exhibit conserved peroxide-mediated cytotoxicity and tumor-specific mechanisms due to their different structural modifications, oncogenic pathways, and microenvironmental factors. Further research is needed to optimize its clinical application.

Although current studies have confirmed the therapeutic efficacy of artemisinin and its derivatives, a comprehensive elucidation of their polypharmacology demands integrating advanced technologies. AI-driven chemoproteomics, longitudinal gut microbiome analysis, and dynamic metabolomics will systematically decode their multi-target mechanisms, particularly in metabolic regulation. Building on this mechanistic foundation, broadening their therapeutic spectrum should explore emerging frontiers—including endocrine disorders (e.g., thyroid dysfunction, adrenal cortex hyperplasia) [127] and cardiovascular pathologies—where their established anti-inflammatory and immunometabolic reprogramming capacities (evidenced in obesity models) may intercept core disease drivers, suggesting untapped potential for chronic inflammatory conditions. For successful clinical translation, two pillars must be prioritized. First, mechanism-focused trials should be designed using cytokine signatures or microbial biomarkers for patient stratification. Second, developing nano-formulations (e.g., lipid-based Pheroid™ systems [257]) and solubility-enhanced delivery platforms [258] can overcome pharmacokinetic limitations, ultimately advancing both therapeutic efficacy and safety.

In summary, shifting artemisinin and its derivatives from a broad-spectrum antimalarial to precision medicine requires a multidisciplinary approach. It integrates multi-omics discoveries, disease-independent mechanism exploration, and intelligent clinical validation frameworks. By prioritizing these directions, the scientific community may fully unlock the revolutionary potential of precision medicine.

## Figures and Tables

**Table 1:** Efficacy of artemisinin and its derivatives on other tumors. “↓” denotes “suppressing or reducing” while “↑” indicates “enhancing or increasing”.

Disease	Compound	Effect	Mechanism	Model	Reference
Leukemia	Dihydroartemisinin	inducing cell death	Mcl-1 ↓, Bcl-2 ↓, Bax ↑, P21 ↑	in vitro	[36]
Ovarian cancer	Artesunate	reducing rRNA transcription, inhibiting cell proliferation and migration, and inducing apoptosis	FANCA-targeted, mTOR ↓, RPS 6 ↓	in vitro and vivo	[90]
Glioblastoma	Dihydroartemisinin	blocking the cell cycle, inducing apoptosis	ERRα ↓, Caspase—3 ↑, Cleaved Caspase—9 ↑, PARP ↑	in vitro and vivo	[91]
	Artesunate	inducing oxidative stress, DNA damage, apoptosis, and ferroptosis	ATM/ATR axis ↑	in vitro and vivo	[92]
Prostate cancer	Dihydroartemisinin	inducing ROS-dependent mitochondrial apoptosis, autophagy	PI3K/AKT/mTOR pathway ↓	in vitro	[93]
Melanoma	Artemisinin	suppressing postoperative recurrence and metastasis	KIT/PI3K/AKT pathway ↓, Cyclin D1 ↓, Bcl-2 ↓	in vitro and vivo	[94]
	Dihydroartemisinin	inducing ROS-dependent apoptosis, inhibiting cell migration	Bcl-2 ↓, Survivin ↓, Bax ↑, MMP—9 ↓	in vitro and vivo	[95]
	Artesunate	inducing ferroptosis, activating CD8^+^ T-cell immunity	IDO1 ↓	in vitro and vivo	[96]
Lymphoma	Artesunate	inducing ROS-dependent apoptosis, ferroptosis, DNA damage, inhibiting angiogenesis, and activating antitumor immunity	Topo I ↓, Topo II ↓	in vitro and vivo	[97]
Gastric cancer	Dihydroartemisinin	inhibiting vasculogenic mimicry	FGF2/FGFR1-MAPK/PI3K pathway ↓, VE-cadherin ↓, MMP-2 ↓, MMP-9 ↓	in vitro and vivo	[98]
Neuroblastoma	Dihydroartemisinin	blocking the cell cycle, inducing mitochondrial apoptosis	PARP-1 ↑, caspase 3 ↑, γH2AX ↑	in vitro	[99]
	Artesunate	blocking the cell cycle, inducing mitochondrial apoptosis	activated caspase-3 ↑, sub-G1 fraction ↑	in vitro	[100]
	Artemether	enhancing the caspase-dependent apoptotic effect of Doxorubicin	B7-H3 ↓, NF-κB pathway ↓	in vitro	[101]
Head and neck squamous cell carcinoma (HNSCC)	Artemisinin	inducing apoptosis, promoting vascular normalization, and enhancing antitumor immunity	MIF ↓, VEGF ↓, IL-8 ↓	in vitro and vivo	[102]
	Dihydroartemisinin	blocking EMT and invasive migration	miR-195-5p ↑, ZEB1/MMP-9 ↓	in vitro	[103]
Cholangiocarcinoma	Dihydroartemisinin	inducing apoptosis and autophagy	DAPK1-BECLIN1 pathway ↑	in vitro	[104]
Osteosarcoma	Dihydroartemisinin	enhancing anti-angiogenesis	Loxl2 ↓, VEGFA ↓	in vitro and vivo	[105]
		blocking the cell cycle	Wnt/β-catenin pathway ↓, Cyclin D1 ↓, c-Myc ↓	in vitro and vivo	[106]
Rhabdomyosarcoma	Dihydroartemisinin	inducing autophagy, blocking the cell cycle	AMPK pathway ↑, mTORC1 ↓	in vitro and vivo	[107]
	Artesunate	inducing mitochondrial apoptosis, inhibiting angiogenesis	ROS ↑, p38 MAPK ↑, VEGF ↓	in vitro and vivo	[38]
Pancreatic cancer	Artesunate	Inhibiting cell growth, inducing apoptosis	Top2A ↓, RRM2 ↓, PCNA ↓, NAG—1 ↑	in vitro	[108]
		inducing ferroptosis	GRP78-xCT axis ↓	in vitro and vivo	[109]
Urothelial carcinoma	Artemisinin	inhibiting proliferation, enhancing the DNA-damaging effects of cisplatin	FGFR3 ↓, HRAS ↓, P53 ↑, KDM6A ↑	in vitro and vivo	[110]
Cervical cancer	Dihydroartemisinin	inducing ferroptosis	GPX4 ↓, SLC7A11 ↓	in vitro	[111]
		inducing apoptosis	RKIP-NF-κB axis ↑, Bcl-2 ↓	in vitro	[112]
		inducing autophagy	Bcl-2 ↓	in vitro and vivo	[113]
		blocking the cell cycle	ROS ↑, caspase-9 ↑, PARP ↑	in vitro and vivo	[114]
	Artesunate	reversing immunosuppression	COX-2 ↓, PGE2 ↓, Foxp3 ↓	in vitro and vivo	[115]
		enhancing the TRAIL-induced DR4/DR5-Caspase Apoptosis	NF-κB ↓, PI3K-Akt ↓	in vitro	[116]
Esophageal carcinoma	Dihydroartemisinin	inhibiting proliferation, promoting apoptosis	DAB2IP ↑, RAS/ERK pathway ↓	in vitro and vivo	[117]
	Artesunate	inducing ferroptosis	AKT/mTOR-xCT/GPX4 axis ↓	in vitro and vivo	[118]
Thyroid cancer	Artemisinin	inhibiting proliferation, promoting apoptosis	XIST/miR-93/HIF-1α axis1 ↓	in vitro and vivo	[119]
Renal carcinoma	Artesunate	inducing ROS-dependent necrotic apoptosis	RIP1-MLKL pathway ↑	in vitro	[120]

**Table 2:** Efficacy of artemisinin and its derivatives on immune disorders. “↓” denotes “suppressing or reducing” while “↑” indicates “enhancing or increasing”.

Disease	Compound	Effect	Mechanism	Model	Reference
Autoimmune hepatitis	TPN10475	reducing serum levels of alanine aminotransferase and aspartate aminotransferase, and inhibiting infiltrating inflammatory T cells	PI3K—AKT pathway ↓, INF-γ ↓, TNF-α ↓	In vivo and in vitro	[166]
	TPN10466	reducing T-cell responses	ERK/JNK/p38 pathway ↓, TNF-α ↓, IL-6 ↓	In vivo	[167]
Autoimmune Encephalomyelitis	TPN10475	increasing Treg, decreasing T cells, and inflammatory cell infiltration	TGF-βpathway ↑, Th1 ↓, Th17 ↓	In vivo and in vitro	[128]
	Dihydroartemisinin	increasing Treg and inhibiting Th cell differentiation	IFN-γ ↓, IL-4 ↓, mTOR pathway ↓, TGF-βR:Smad pathway ↑	In vivo and in vitro	[168]
Melanoma	Dihydroartemisinin	increasing CTL, inhibiting the immunosuppressive effect of Treg, and the invasion and migration of tumor cells	STAT3 pathway ↓, Treg ↓, IL-10 ↓	In vivo and in vitro	[169]
Sjögren’s syndrome	Artesunate	reducing lymphocyte infiltration and immune inflammation	IRF4 ↓, Th17 ↓	In vivo and in vitro	[170]
Atherosclerosis	Artemisinin	inhibiting foam macrophage transformation and enhancing macrophage autophagy	AMPK pathway ↑, mTOR ↓, ULK1 ↓, LC—3II ↑, P62 ↓	In vivo	[171]
	Artesunate	reducing the size of atheroplaque and lipid deposition	KLF2/NRF2/TCF7L2 pathway ↑, LPL ↑	In vivo	[172]
Acute lung injury	Dihydroartemisinin	inhibiting LPS-induced inflammatory cell infiltration, oxidative stress, and pro-inflammatory cytokine production	IL-1β ↓, TNF-α ↓, IL-6 ↓, NF-κB pathway ↓, Nrf2 pathway ↑	In vivo and in vitro	[173]
Ulcerative colitis (UC)	Dihydroartemisinin	reducing inflammatory cell infiltration	JAK2/STAT3 pathway ↓, IL-6 ↓, IL-1β ↓, TNF-α ↓	In vivo	[174]
	Artemether	inhibiting ROS production and preventing the assembly and activation of NLRP3 inflammasomes	IL-1β ↓, IL-6 ↓, TNF-α ↓, mtROS ↓	In vivo and in vitro	[175]
Dry eye disease	SM934	reducing inflammatory cell infiltration and inflammatory factor expression	IL-1β ↓, IL-6 ↓, TNF-α ↓, TLR4/NF-κB pathway ↓, NLRP3 ↓	In vivo	[140]

**Table 3:** Efficacy of artemisinin and its derivatives on metabolic disorders. “↓” denotes “suppressing or reducing” while “↑” indicates “enhancing or increasing”.

Diseases	Compound	Effect	Mechanism	Progress	References
Type 2 diabetes	Artemisinin	reducing blood glucose, oxidative stress, and inflammation levels and reversing IR; increasing regeneration of pancreatic β-cell	Pax4 ↑, Arx ↑	in vitro and in vivo	[181]
	Artemether	promoting glycogen synthesis with antidiabetic effects	AMPK ↑, PI3K/Akt ↑	in vivo	[183]
Diabetic cardiomyopathy	Artemisinin	reducing oxidative stress, inflammation, and fibrosis	AGE-RAGE/HMGB-1 ↓	in vivo	[193]
Leukemia	Dihydroartemisinin	inhibiting leukemia cell proliferation by regulating glycolysis and metabolism	PKM2 ↓, GLUT1 ↓	in vitro	[184]
Non-small-cell lung cancer	Dihydroartemisinin	inhibiting glucose metabolism via NF-κB pathway inhibition	NF-κB↓, GLUT1↓	in vitro and in vivo	[191]
Obesity	Artesunate	reducing food intake and promoting energy expenditure, leading to weight loss	GDF15/GFRAL ↑	in vivo	[197]
	Artesunate	inhibiting lipid accumulation and TG synthesis	C/EBP-a ↓, PPAR-γ ↓, FAS ↓, periilipin A ↓, STAT-3 ↓	in vitro	[195]
Atherosclerosis	Artemisinin	reducing ROS and NO; suppressing lipid influx; reducing the uptake and internalization of oxLDL	NF-κB/NLRP3 ↓, AMPK/mTOR ↑	in vitro and in vivo	[202]
	Artemisinin	suppressing inflammatory reaction	NF-κB/NLRP3 ↓, AMPK ↑	in vivo	[204]
	Artesunate	reducing pro-inflammatory cytokine and downregulating the pro-inflammatory chemokines; attenuating the progression of atherosclerosis lesions	TNF-a ↓, IL-6 ↓, IL-8 ↓, MCP-1 ↓	in vivo	[194]
Fatty liver diseases	Artemether	inhibiting microglial activation in the hypothalamus; protecting the TRH neurons from inflammatory damage and promoting the release of TRH	palmitoylation of PKCδ ↓	in vivo	[205]
Obesity-related glomerulopathy	Artemisinin	reducing the degradation of vitamin D; mitigating renal tubular damage and fibrosis	CYP24A1 ↓, vitamin D ↑	in vivo	[206]
Nonalcoholic steatohepatitis (NASH)	Dihydroartemisinin	decreasing the synthesis of lipids; inhibiting OS and ERS; reducing liver steatosis	NF-κB ↓	in vivo	[207]
Thyroid eye disease (TED)	Dihydroartemisinin, Artesunate	inhibiting adipogenesis; inhibiting cell proliferation and hyaluronan production	HAS2 ↓, IGF1R ↓, adipogenic markers ↓	in vitro	[208]
Amiodarone-induced pulmonary toxicity	Artemisinin	regulating cellular energy homeostasis and mitigating oxidative stress; protecting against amiodarone-induced apoptosis and bronchial epithelial cell shedding	p-AMPK ↑, CaMKK2 ↑, Nrf2 ↑, SOD1 ↑	in vitro and in vivo	[209]
Amiodarone-induced ocular toxicity	Artemisinin	reducing the cytotoxicity induced by amiodarone	LDH ↓, ROS ↓, MMP ↑, p-AMPK ↑, CaMKK2 ↑, Nrf2 ↑	in vitro	[210]
APAP-induced acute liver injury (AILI)	Artemether	mitigating liver histological changes, including mitochondrial damage, hepatocyte necrosis, hepatocyte apoptosis, and inflammatory infiltration	Nrf2-HO-1/GPX4 ↑, ROS ↓	in vivo	[211]
Abnormal white matter development (periventricular leukomalacia)	Artemisinin	reducing perinatal inflammation and inflammatory cytokine production	IRAK-4 ↓, IRAK-1 ↓, IL-1β ↓, IL-6 ↓, TNF-α ↓, ROS ↓, Nrf2 ↑	in vitro	[212]
Periprosthetic osteolysis (PPO)	Artemisic acid	reducing osteoclast formation and alleviating titanium particle-induced calvarial osteolysis	ROS ↓, Nrf2 ↑, MAPK ↓, NF-κB ↓, NFATc1 ↓, c-Fos ↓	in vitro and in vivo	[186]
Polycystic ovarian syndrome (PCOS)	Artemisinin	inhibiting ovarian androgen synthesis; mediating LONP1-CYP11A1 interaction; regulating mitochondrial function	CYP11A1 ↓	in vitro and in vivo	[213]

## Abbreviations

The following abbreviations are used in this manuscript:

CDK	Cyclin-dependent kinase
p16	Cyclin-dependent kinase inhibitor 2A (CDKN2A)
ERK1/2	Extracellular signal-regulated protein kinase 1/2
CDK4	Cyclin-dependent kinase 4
CDK2	Cyclin-dependent kinase 2
RB	Retinoblastoma protein
TOM70	Translocase of Outer Mitochondrial Membrane 70
P450	Cytochrome P450
XO	Xanthine Oxidase
Cytc	Cytochrome c
ROS	Reactive Oxygen Species
RTK-i	Receptor Tyrosine Kinase inhibitor
Nrf2	Nuclear factor erythroid 2-related factor 2
MAPK	Mitogen-activated protein kinase
Src	Proto-oncogene tyrosine-protein kinase Src
NF-κB	Nuclear factor kappa-light-chain-enhancer of activated B cells
PI3K/Akt	Phosphoinositide 3-kinase—Protein kinase B
MMP	Matrix metalloproteinases
Met	Hepatocyte Growth Factor Receptor
Rho	Ras homolog
Rac	Ras-related C3 botulinum toxin substrate
TfR1	Transferrin Receptor 1
SLC3A2	Solute Carrier Family 3 Member 2
SLC7A11	Solute Carrier Family 7 Member 11
c-Casp9	Cleaved Caspase-9
c-Casp3	Cleaved Caspase-3
c-PARP	Cleaved Poly (ADP-ribose) Polymerase
PUFA-PL	Polyunsaturated Fatty Acid-Phospholipid
ALOXs	Arachidonate lipoxygenases
POR	Cytochrome P450 oxidoreductase
PUFA-PL-OOH	Polyunsaturated Fatty Acid-Phospholipid Hydroperoxide
PINK1	PTEN Induced Kinase 1
PARK2	Parkin RBR E3 Ubiquitin Protein Ligase
TLR	Toll-like Receptor
PI3K	Phosphoinositide 3-Kinase
AKT	Protein Kinase B (PKB)
c-Fos	Cellular FOS Proto-Oncogene
IFN-γ	Interferon-Gamma
TNF-α	Tumor Necrosis Factor-α
BTK signal	Bruton’s Tyrosine Kinase signaling
Myc	MYC Proto-Oncogene
AMPK	AMP-activated Protein Kinase
mTOR	Mechanistic Target of Rapamycin
ULK1	Unc-51 Like Autophagy Activating Kinase 1
VDR	Vitamin D Receptor
HIF-1α	Hypoxia-Inducible Factor 1-α
NLRP3	NLR Family Pyrin Domain Containing 3
GLUT1	Glucose Transporter 1
GSK-3β	Glycogen Synthase Kinase 3 beta
β3	Integrin beta-3
MCP-1	Monocyte Chemoattractant Protein-1
NOX4	NADPH Oxidase 4
PPARα	Peroxisome Proliferator-Activated Receptor α
Klf2	Krüppel-Like Factor 2
LPL	Lipoprotein Lipase
ABCA1	ATP-Binding Cassette Subfamily A Member 1
TG	Triglyceride
TC	Total Cholesterol
